# Numerical Investigation of Polymer Coated Nanoporous Gold

**DOI:** 10.3390/ma12132178

**Published:** 2019-07-06

**Authors:** Stephan Gnegel, Jie Li, Nadiia Mameka, Norbert Huber, Alexander Düster

**Affiliations:** 1 Numerical Structural Analysis with Application in Ship Technology (M-10), Hamburg University of Technology, Am Schwarzenberg-Campus 4 (C), 21073 Hamburg, Germany; 2 Institute of Materials Research, Materials Mechanics, Helmholtz-Zentrum Geesthacht, Geesthacht 21502, Germany; 3 Institute of Materials Physics and Technology, Hamburg University of Technology, 21073 Hamburg, Germany

**Keywords:** nanoporous gold, polymer coating, finite cell method, window method

## Abstract

Nanoporous metals represent a fascinating class of materials. They consist of a bi-continuous three-dimensional network of randomly intersecting pores and ligaments where the ligaments form the skeleton of the structure. The open-pore structure allows for applying a thin electrolytic coating on the ligaments. In this paper, we will investigate the stiffening effect of a polymer coating numerically. Since the coating adds an additional difficulty for the discretization of the microstructure by finite elements, we apply the finite cell method. This allows for deriving a mesh in a fully automatic fashion from the high resolution 3D voxel model stemming from the 3D focused ion beam-scanning electron microscope tomography data of nanoporous gold. By manipulating the voxel model in a straightforward way, we add a thin polymer layer of homogeneous thickness numerically and study its effect on the macroscopic elastic properties systematically. In order to lower the influence of the boundary conditions on the results, the window method, which is known from homogenization procedures, is applied. In the second part of the paper, we fill the gap between numerical simulations and experimental investigations and determine real material properties of an electrolytic applied polypyrrole coating by inverse computations. The simulations provide an estimate for the mechanical properties of the ligaments and the polymeric coating and are in accordance with experimental data.

## 1. Introduction

Nanoporous gold (NPG) represents a fascinating class of nanoporous metals. An overview over their morphologies and mechanical properties can be found, for example, in [1,2] and [3,4]. NPG consists of a three-dimensional network of randomly intersecting pores. Ligaments form the skeleton of the structure. Their diameter can be controlled by altering dealloying conditions, thus allowing for examining the impact of the ligament size on the macroscopic mechanical properties [5,6,7,8].

The open-pore structure of NPG enables creating gold–polymer composites (NPG-composites) either by fully infiltrating the porous metal by epoxy resins [9,10] or by adding a thin electrolytic coating layer of a conjugated polymer (such as polyaniline or polypyrrole) on top of the ligaments [11,12,13,14]. The latter strategy has resulted in novel hybrid materials with improved mechanical behavior along with substantially enhanced functional properties [14]. The suggested potential application of such nanocomposites as mechanical actuators requires a detailed understanding of the impact of the polymer layer on the effective mechanical response. This study thus aims to elucidate the effects of a thin polymeric coating on the macroscopic stiffness of the NPG-composites numerically. However, finite element modeling of NPG alone is challenging due to its complex 3D network structure. The existing literature can be categorized in models based on simplified unit cells with defined coordination number and ligament geometry [15,16,17,18,19,20,21,22,23], artificial structures generated with the help of physical or semi-physical models for a given solid fraction [22,24,25,26,27], and models that are derived from 3D tomography data obtained from nanoporous gold samples [28,29]. Thus far, the simulation of the elastic-plastic deformation behavior is essentially restricted to the approaches making use of Finite Element Method (FEM) beam models, which are on the one hand highly efficient, but have on the other hand some limitations concerning the quantitative prediction of macroscopic properties due to the effect of the nodal masses in the connecting nodes [15,17,21]. Motivated by this, a nodal corrected beam model is developed that corrects the softening inherent in the FEM beam model by a parametrization of the diameter and Young’s modulus of the beam elements in the region of the nodal mass [18]. This nodal corrected beam model is able to predict the stress–strain behavior up to more than 10% strain with high computational efficiency. However, the parametrization for the nodal correction is so far only available for ball-and-stick models with cylindrical ligaments connected in spherical nodal masses. As the parametrization of the nodal beam elements depends on the local geometry of the ligament and the nodal mass, a generalization towards concave, convex, and asymmetric ligament shapes reported by [29] is required for applying such a nodal correction to realistic structures. Furthermore, it is shown in [29] that FEM beam modeling of realistic structures, based on skeletonization of 3D tomography data, can result in a systematic and significant overestimation of stiffness and strength. This is due to the nature of the thickness algorithm of [30] implemented in the open-source software FIJI, which overestimates the diameters of non-cylindrical ligaments. Lacking a sufficiently accurate approach for the thickness determination and a general method for the nodal correction, a straightforward translation of 3D tomography data into a 3D FE model is highly desirable. As shown in [28], it is possible to build a 3D solid model from 3D tomography data that allows for an elastic-plastic simulation with affordable computational effort. However, more work needs to be invested towards the discretization and the boundary conditions that deal with the limited size and the non-periodic nature of representative volume elements (RVEs) as well as the spatial resolution of nanosized features of such highly complex structures. Furthermore, conventional 3D meshing has substantial limitations in adding a thin coating to such a geometry. Therefore, we apply the finite cell method (FCM) to investigate the effect of a polymer coating on the macroscopic elastic-plastic response. In contrast to other discretization methods, simulations can be performed directly on a high-resolution 3D voxel model stemming from the 3D focused ion beam-scanning electron microscope (FIB-SEM) tomography data of an NPG sample, provided by [28]. To deal with the limited RVE size and the non-periodic nature which does not permit to use symmetry boundary conditions, we apply the window method which applies the boundary conditions in a self-consistent way. By inverse computations of NPG composites where the epoxy resin and polypyrrole (PPy) were exploited as polymeric phases, we aim to obtain important insights in the mechanical properties of thin PPy coatings deposited on NPG.

## 2. Experimental Investigations

Nanoporous Au-polypyrrole (NPG-PPy) composites were prepared and tested mechanically. This section briefly describes the sample preparation procedure and the subsequently performed mechanical tests.

### 2.1. Preparation of Nanoporous Au-Polypyrrole (NPG-PPy) Composites

**Figure 1:**
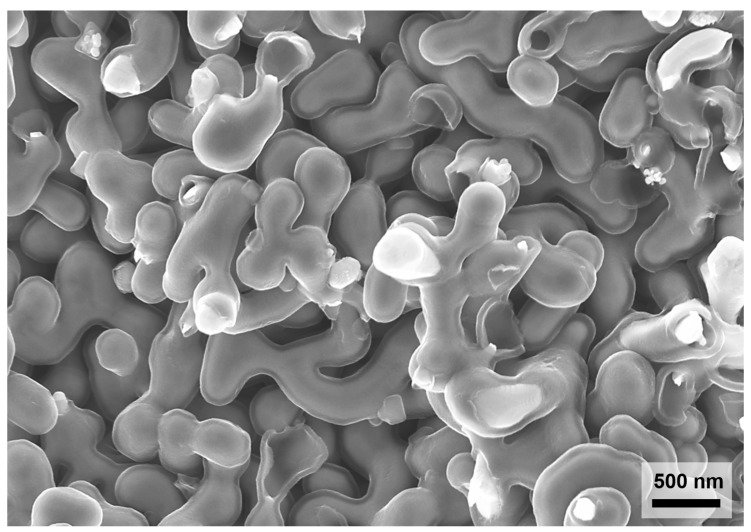
Scanning electron microscope micrograph of nanoporous gold (NPG) with ligament diameter about 220 nm coated by a polypyrrole (PPy) layer of 50 nm. Note that the nanoporosity is preserved after the electrodeposition of the PPy coating on the gold ligaments.

The NPG samples were prepared by electrochemical dealloying of a $Au_{25}Ag_{75}$ precursor alloy. The used established procedures are described in detail in [31]. The master alloy ingot was prepared by arc melting (arc melter MAM-1, Edmund Bühler) and homogenized at $800{\;}^{\circ }C$ for 120 h (furnace RHF1600, Carbolite) by sealing it in an evacuated (≈$10^{-2}\;\mathrm{bar}$) quartz tube. This was followed by wire drawing and cutting of the ingot to fabricate cylindrical specimens with dimensions of 1.2mm×1.5mm, which were subsequently annealed in argon atmosphere for recovery (2 h at $800{\;}^{\circ }C$, infrared furnace behr IRF 10, behr Labor Technik, Düsseldorf, Germany). The dealloying was carried out in a three-electrode electrochemical cell at a constant potential 0.75V vs. a Ag/AgCl pseudo reference electrode in 1 M $HClO_{4}$ electrolyte (60% $HClO_{4}$, ACS grade, Merck, Darmstadt, Germany) at room temperature. A coiled Ag wire (99.9985%, Alfa Aesar, Kandel, Germany) was used as the counter electrode. Afterwards, the as-dealloyed samples were electrochemically reduced by cyclic voltammetry cycles (15 cycles within a potential range of −0.5V…1.0V vs. the Ag/AgCl pseudo in 1 M $HClO_{4}$ prepared from Suprapur grade HClO4, Merck), rinsed with ultrapure water, and dried in air. PPy was deposited into bulk NPG specimens by the electropolymerization of pyrrole monomer (pyrrole, 99%, extra pure, ACROS Organics) in a lithium perchlorate acetonitrile solution solution ($LiClO_{4}$, 99.99%, Sigma-Aldrich, Darmstadt, Germany, acetonitrile, LiChrosolv, Merck), following a synthesis protocol described in [14]. The resulting PPy was thus doped with $ClO_{4}^{-}$. Before the electropolymerization, NPG was annealed in air ($500{\;}^{\circ }C$ for 30 min) to coarsen the pores. This was necessary to facilitate diffusion of pyrrole monomers in the porous network in order to achieve a uniform PPy coating throughout the bulk NPG samples. To obtain a PPy layer with a predefined thickness and to keep the nanoporosity, the total electrodeposition time (800 s) was optimized accordingly. All electrochemical measurements were performed using a potentiostat PGSTAT 302N (Metrohm). Finally, the NPG-PPy composites were cleaned with ultrapure water and dried at ambient conditions. Figure 1 shows a typical microstructure of the resulting NPG-PPy composites after the electropolymerization (characterized by a high resolution scanning electron microscope Zeiss Supra 55VP FEG SEM, Jena, Germany). Investigation of the fracture surfaces of the PPy-coated NPG (obtained through cleavage with a scalpel) revealed an average ligament size of ≈220±30nm and ≈50±8nm PPy layer. ImageJ (version 1.49v, National Institutes of Health, Bethesda, MD, USA) was used to analyze the sizes of gold ligaments and PPy coatings in SEM images by averaging 20 measurements.

### 2.2. Compression Tests

Before uniaxial compression with several unloading-reloading sequences were undertaken, the NPG and PPy-coated NPG bulk specimens were cleaned and dried. Afterwards, they were subjected to a similar testing protocol as employed in [15]. Linear segments of the first unload–reload cycles were used to compute the effective Young’s moduli. The mechanical experiments were carried out on a calibrated universal testing machine *Zwick Z010 TN, (Ulm, Germany)* at a constant engineering strain rate of $10^{-4}s^{-1}$ and ambient conditions. A sample displacement tracking in a longitudinal direction during compression was achieved by a laser extensometer (*Zwick laserXtens*). The displacement data served to determine the true strains. We presented the experimental stress–strain diagrams in true stress-true strain coordinates. Because of the insignificant cross-section area variation of NPG upon compressive loading [32,33], engineering and true stresses practically coincide.

## 3. Modeling and Simulation of Nanoporous Gold Based on the Finite Cell Method

This section is intended to give a short introduction into the FCM which was applied for the simulation of NPG. A subsection is related to the window method that describes the approach to find homogenized elastic properties for the NPG samples.

The finite cell method [34,35] relies on the fictitious domain approach in combination with the high-order finite element method. The well known weak form of the equilibrium conditions in the current configuration reads:

(1) $$g\left(\varphi ,\eta \right)=\int_{\Omega }\sigma \;\cdot \;\mathrm{grad}\;\eta \;dv-\int_{\Omega }\rho b\;\cdot \;\eta \;dv-\int_{\Gamma_{N}}t\;\cdot \;\eta \;da=0,$$

where φ describes the mapping between the reference and the current configuration, σ is the Cauchy stress tensor, b denotes the volumetric loads and t the prescribed traction acting on the Neumann boundary $\Gamma_{N}$ of the body Ω. The current density is denoted by ρ and η are the test functions or virtual displacements.

After introducing the indicator function α and reformulating Equation (1) for an extended domain $\Omega_{e}$, we obtain the following form which is the basis for the FCM:

(2) $$g\left(\varphi ,\eta \right)=\int_{\Omega_{e}}\alpha \;\sigma \;\cdot \;\mathrm{grad}\;\eta \;dv-\int_{\Omega_{e}}\alpha \;\rho b\;\cdot \;\eta \;dv-\int_{\Gamma_{N}}t\;\cdot \;\eta \;da=0.$$

**Figure 2:**
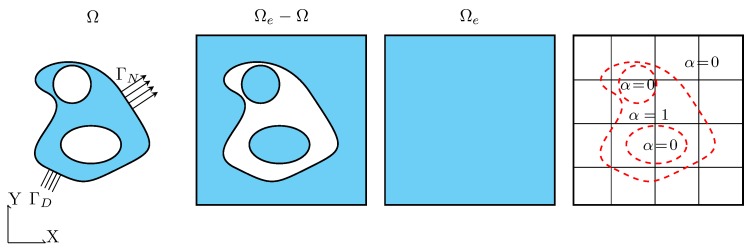
The concept of the Finite Cell Method is to embed the physical domain Ω into a bigger domain that can be easily meshed.

The modified weak form allows to extend any arbitrarily complex shaped geometry by a fictitious domain $\Omega_{f}\left(\Omega_{e}-\Omega \right)$ such that it obtains a simple shape. In general, this is just the bounding box of the underlying geometry. Now, a simple discretization of the extended domain $\Omega_{e}$ can be derived by a Cartesian grid that does not conform with the boundary of the original problem—see Figure 2.

The equivalence with the original problem is enforced by the discontinuous indicator function:

(3) $$\alpha \left(X\right)=\left\{\begin{array}{cc} 1, & \;\forall X\in \Omega ,\\ \alpha_{0}=10^{-q}, & \;\forall X\in \Omega_{e}-\Omega \end{array}\right.$$

that penalizes the fictitious domain. Both formulations are equivalent for q=∞ but in fact we choose q∈[5,…,12] to avoid ill-conditioning of the problem. By this little modification, the effort of mesh generation is considerably reduced due to the rectangular shape of the extended domain and shifted towards the integration of the weak form that involves a discontinuity caused by the indicator function. The quadrature of the weak form can be carried out by an adaptive Gaussian quadrature scheme in a straightforward and efficient way that only involves point-membership-tests based on the underlying geometric models. To this end, every integration point has to be tested whether it lies in the physical domain. This feature makes the FCM flexible so that it can directly operate on implicitly defined geometries, i.e., voxel-based data generated by computer tomography or B-rep models [36].

### 3.1. Adaptive Gaussian Quadrature

**Figure 3:**
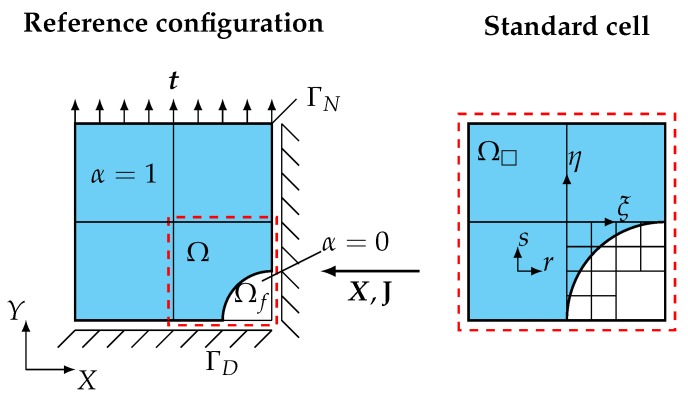
Adaptive Gaussian integration and quadtree refinement scheme.

The discretization of the weak form in Equation (2) leads to a set of finite cells that can be classified into three groups. Cells that are completely placed within the fictitious domain do not contribute to the weak form (α=0) and can be disregarded. For cells fully lying in the physical part of the domain (full cells) α=1 holds, so they are equivalent to standard finite elements and can be integrated in the standard fashion. Special care has to be taken for cells placed over the boundary of the original domain (cut cells) that exhibit a discontinuous integrand due to the indicator function α. The adaptive Gaussian quadrature scheme subdivides the integration domain $\Omega_{□}$ of these cells recursively along the discontinuity into smaller sub-cells $\Omega_{sc}$ (Figure 3) until a predefined refinement level is reached. The integrand can then be computed as a composed Gaussian quadrature on the level of the sub-cells:

(4) $$\int_{\Omega_{□}}\left(\;\cdot \;\right)\alpha \left(X(\xi )\right)\det J\left(\xi \right)d\Omega_{□}=\sum_{sc=1}^{n_{c}}\sum_{i=1}^{n_{G}}\left(\;\cdot \;\right)\alpha \left(X(\xi \left(r_{i}\right))\right)\det J\left(\xi \left(r_{i}\right)\right)\det J^{sc}\left(r_{i}\right)\;\omega_{r_{i}},$$

where $r_{i}$ are the well-known Gauss–Legendre points and $\omega_{i}$ the corresponding weights.

To organize the refinement process space trees are used, e.g., quadtrees in 2D (Figure 3) and octrees in 3D problems. Since $\alpha \left(X(\xi \left(r_{i}\right))\right)\det J^{sc}\left(r_{i}\right)\;\omega_{r_{i}}$ and $\xi \left(r_{i}\right)$ are constant during the computation, they can be stored as the resulting weights and coordinates of the integration points belonging to the adaptive integration scheme. When considering nonlinear problems, the solution of the nonlinear set of algebraic equations by means of the Newton–Raphson method requires the linearization with respect to the unknown displacements Δu—see, for example, [37]. To simplify the notation, we do not take follower loads and volume loads into account. The resulting linear equation system that is solved within each iteration *k* for every loadstep *i* reads:

(5) $$K^{k-1}\Delta u_{i}^{k}=f_{\mathrm{int}}^{k-1}\left(u_{i}^{k-1}\right)-\bar{\lambda_{i}}f_{\mathrm{ext}},$$

where $K^{k-1}$ is the global tangent stiffness matrix that stems from the discretization of the linearization of the weak form and $\bar{\lambda_{i}}$ is the accumulated load factor which serves to apply the load increment-wise. The global matrices are computed by assembling the tangent stiffness matrix and the load vector of every cell *c*:

(6) $$K=\overset{n_{c}}{\underset{c=1}{A}}K_{c}\;f_{\mathrm{int}}=\overset{n_{c}}{\underset{c=1}{A}}f_{\mathrm{int},c}\;f_{\mathrm{ext}}=\overset{n_{c}}{\underset{c=1}{A}}f_{\mathrm{ext},c},$$

where the tangent stiffness matrix, the internal and the external force vector of cell *c* read:

(7) $$K_{c}=\int_{\Omega_{□}}\;B^{T}\alpha CB\;\det J\;d\Omega_{□}\;f_{\mathrm{int},c}=\int_{\Omega_{□}}\;B^{T}\alpha \sigma \;\det J\;d\Omega_{□}\;f_{\mathrm{ext},c}=\int_{\Gamma_{N,c}}\;N^{T}t\;∥q_{,\alpha }\times q_{,\beta }∥\;d\alpha d\beta \;.$$

Here, B presents the strain–displacement matrix containing the derivatives of the high-order shape functions, and C is the spatial elasto-plastic tangent modulus that is consistent with the radial return mapping scheme applied to integrate the elastoplastic material model. The computation of the external load vector $f_{\mathrm{ext}},c$ requires integrating the product of the shape functions and the traction vector over the surface of the corresponding cell. In Equation (7), $q_{,\alpha }$ and $q_{,\beta }$ denote the tangent vectors of the surface of the cell in question, where α and β are two of the three local coordinates of the related cell.

### 3.2. Von Mises Plasticity

The mathematical formulation of plasticity [38] in the small strain case relies on an additive split of the small strain tensor ε into an elastic $\varepsilon^{e}$ and a plastic contribution $\varepsilon^{p}$:

(8) $$\varepsilon =\varepsilon^{e}+\varepsilon^{p}.$$

The constitutive relation for the stress is given by the derivative of the strain energy function ψ with respect to the elastic strains $\varepsilon^{e}$:

(9) $$\sigma =\rho \frac{\partial \psi }{\partial \varepsilon^{e}}=\lambda \mathrm{tr}\;\left(\varepsilon^{e}\right)1+2\mu \varepsilon^{e},$$

where λ,μ are the *Lamé constants* and 1 is the second order identity tensor. In order to define the onset of yielding, we use the von Mises yield function:

(10) $$\Phi =\sqrt{3J_{2}\left(s\right)}-\sigma_{y}\left({\bar{\varepsilon }}^{p}\right)\leq 0,$$

where s is the deviatoric part of the stress tensor and $J_{2}$ denotes its second invariant.

The evolution of the plastic strains is described by the equation:

(11) $${\dot{\varepsilon }}^{p}=\dot{\gamma }\frac{\partial \Phi }{\partial \sigma },$$

where $\dot{\gamma }$ is the incremental plastic multiplier.

The hardening variable ${\bar{\varepsilon }}^{p}$ is related to the incremental plastic strain by

(12) $${\bar{\varepsilon }}^{p}=\int_{0}^{t}\sqrt{\frac{2}{3}}∥{\dot{\varepsilon }}^{p}∥dt\;.$$

### 3.3. The Window Method

**Figure 4:**
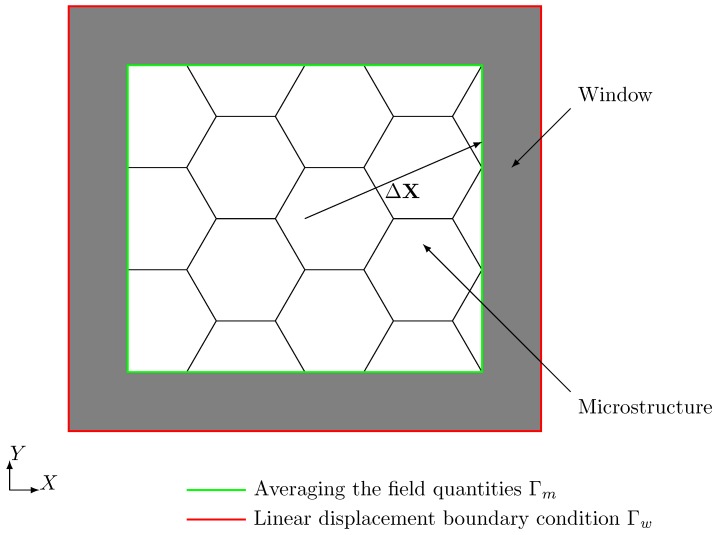
The window with embedded microstructure.

The computation of the effective material properties for heterogeneous materials calls for a high computational effort due to the requirement of large representative volume elements (RVE). Therefore, the analysis of such structures is only possible for smaller testing volume elements (TVE) and thus the influence of boundary conditions on the results is large. The *window method* [39] lowers this influence by embedding the TVE into a domain (window) with homogeneous material and applying displacement boundary conditions to the surface of the window as depicted in Figure 4.

This approach is known from analytical homogenization schemes and called the *self-consistency method*—see [40]. The window models the surrounding material that a real RVE would be embedded in and reduces the influence of the boundary conditions on the results such that tighter bounds for the effective properties are obtained—see [41].

In order to achieve *self-consistency*, the window material has to have the same effective material properties as the embedded microstructure. It is unknown at the beginning of the computations and therefore an iterative procedure is required to determine the effective material properties and adjust the material of the window in every step.

In the following, we assume that the material behavior is linear elastic and inclusions or coatings are perfectly bonded to the surrounding material. An anisotropic material requires solving six linear independent loadcases j=1,…,6 where every loadcase determines one column of the elasticity matrix. To this end, we define the macroscopic small strain projection tensor $\varepsilon_{P}$ and disturb a single component of it in each loadcase by a small increment. The projection rule:

(13) $$\Delta {\bar{u}}_{j}=\varepsilon_{P}\;\Delta X\;$$

imposes the related displacements $\Delta \bar{u}$ on the surface of the window $\Gamma_{w}$. Here, ΔX is the branch vector that points from the center of the TVE to the location of the applied displacements. After solving the mechanical problem, the averaged stresses ${\langle \sigma \rangle }_{m}$ and strains ${\langle \varepsilon \rangle }_{m}$ are computed in a post-processing step. The discrete form of the averaged stress reads:

(14) $${\langle \sigma \rangle }_{m}=\frac{1}{V_{m}}\sum_{i=1}^{n_{nodes}}r_{m}^{\left(i\right)}⊗\Delta X^{\left(i\right)},$$

where ⊗ is the dyadic product and $r_{m}^{\left(i\right)}$ are the nodal forces related to the nodes $n_{nodes}$ on the surface $\Gamma_{m}$ of the spatial discretization of the microstructure. The computation of the average stress in Equation (14) might yield an unsymmetric stress tensor which is therefore symmetrized in a subsequent step. The computation of the averaged strains in its discrete form reads:

(15) $${\langle \varepsilon \rangle }_{m}=\frac{1}{2\;V_{m}}\sum_{i=1}^{n_{nodes}}\left(u_{m}^{\left(i\right)}⊗n_{m}^{\left(i\right)}+n_{m}^{\left(i\right)}⊗u_{m}^{\left(i\right)}\right)\;A_{m}^{\left(i\right)},$$

where $u_{m}^{\left(i\right)}$ are the nodal displacements and $n_{m}^{\left(i\right)}$ the surface normal vector. In Equations (14) and (15), $V_{m}$ represents the volume of the microstructure and $A_{m}$ is the inner surface area of the window.

After solving a loadcase one column of the effective elasticity matrix, $C^{\mathrm{eff}}$ is obtained by numerical differentiation of the averaged stress quantities with respect to the related applied macroscopic strains. Applying the Voigt notation for the strains and stresses, the effective elasticity matrix reads:

(16) $$\begin{array}{c} C_{ij}^{\;\mathrm{eff}}=\left\{\begin{array}{cc} \frac{\langle \sigma_{1}^{\;i}\rangle -\langle \sigma_{j+1}^{\;i}\rangle }{{∥\langle \varepsilon_{1}\rangle -\langle \varepsilon_{j+1}\rangle ∥}_{L_{2}}} & \mathrm{if}j\leq 3,\\ \frac{\langle \sigma_{1}^{\;i}\rangle -\langle \sigma_{j+1}^{\;i}\rangle }{2{∥\langle \varepsilon_{1}\rangle -\langle \varepsilon_{j+1}\rangle ∥}_{L_{2}}} & \mathrm{if}j>3, \end{array}\right. \end{array}$$

where *i* corresponds to the stress component and j=1,…,6 denotes the corresponding load case. The basic load case is denoted by index 1 and can be related to a case with no loads.

**Figure 5:**
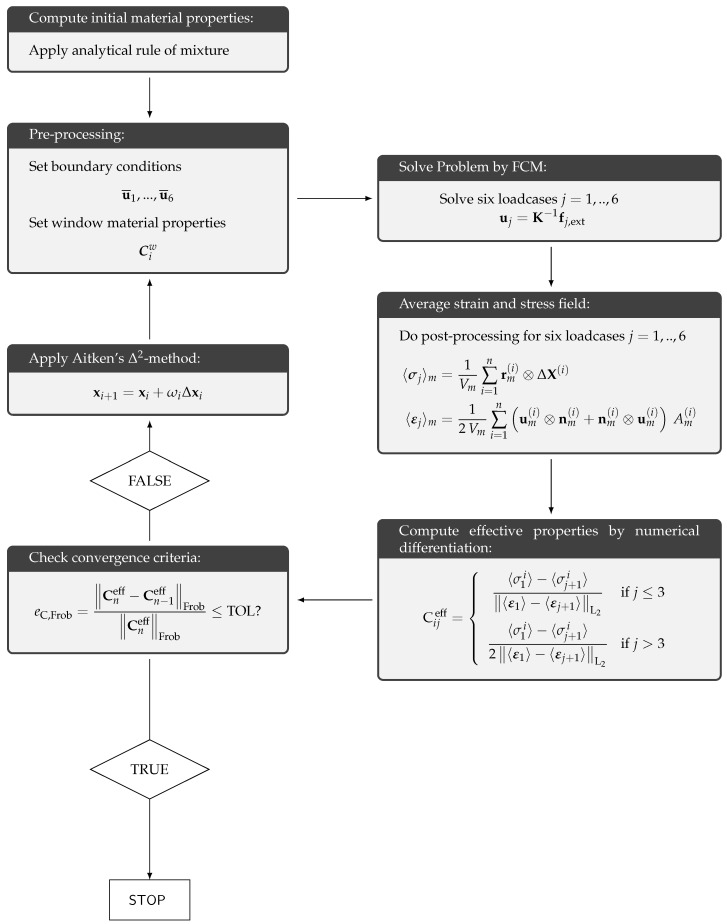
The homogenization procedure.

In order to obtain a symmetric elasticity matrix, we again symmetrize $C^{\mathrm{eff}}$. To adjust the window material in a self-consistent way, the elasticity matrix of the window $C^{w}$ is updated for the next iteration by the actual computed effective properties [42]—see Figure 5. The iterative procedure can be accelerated by the *Aitken’s* $\Delta^{2}$-method [43] and is considered to be converged when the difference between the effective properties of two consecutive steps falls below a prescribed tolerance $e_{C,\mathrm{Frob}}\leq \mathrm{TOL}$ in terms of the *Frobenius* norm,

(17) $$e_{C,\mathrm{Frob}}=\frac{{∥C_{n}^{\mathrm{eff}}-C_{n-1}^{\mathrm{eff}}∥}_{\mathrm{Frob}}}{{∥C_{n}^{\mathrm{eff}}∥}_{\mathrm{Frob}}}.$$

## 4. Numerical and Experimental Investigation of the Influence of Coating on Nanoporous Gold

This section is separated into two parts. In the first part of this section, we investigate the geometrical stiffening effect caused by a polymeric coating. To this end, we consider macroscopic samples of nanoporous gold with and without a coating and analyze it under the assumption that we can apply bulk material values for the gold ligaments and the polymer coating. To find out the orthotropic material parameters of such samples and to quantify the stiffening effect caused by the coating, we perform a homogenization by means of the window method.

The second part of this section is related to the comparison of numerical computations on NPG-PPy-composites with a ligament diameter of $d_{lig}=421\;nm$ and a coating thickness of $t_{c}=107.16\;nm-138\;nm$ to experimental investigations on NPG-PPy samples with a ligament diameter of $d_{lig}=220\;nm$ and a PPy coating thickness of $t_{c}=50\;nm$. Under the assumption of self-similarity of these structures, which was shown in [28,44], we aim to solve the inverse problem, meaning to find the material parameters of the polymer coating such that numerically and experimentally obtained stress–strain curves coincide. The self-similarity properties of NPG were also studied in [45].

### 4.1. Linear Elastic Properties

**Figure 6:**
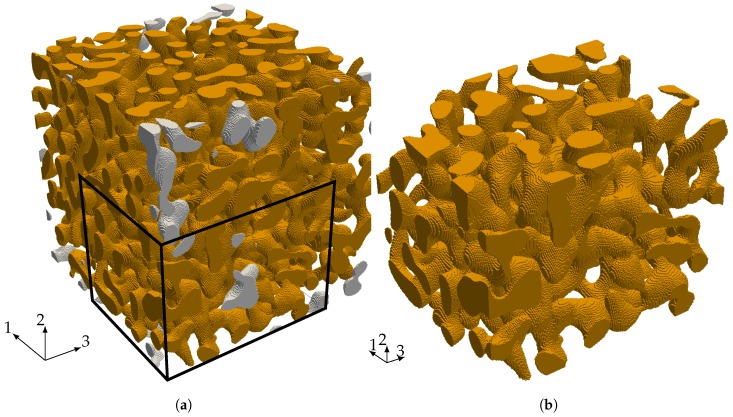
(**a**) Representative Volume Element (RVE) of nanoporous gold $d_{lig}=421\;nm$ of size 5.98μm×5.98μm×5.98μm with 335×335×260 voxels recorded with a resolution of 17.86nm×17.86nm×23nm and (**b**) a sub-sample with a size of 3.57μm×3.57μm×4.6μm with 200×200×200 voxels extracted from the full model.

The starting point for our investigation of the elastic properties of nanoporous gold with a mean ligament diameter of $d_{lig}=421\;nm$ is a voxel model of a representative sample—see Figure 6a. The data was obtained by 3D focused ion beam-scanning electron microscope (FIB-SEM) tomography undertaken by [28,46]. The solid fraction of the sample is 32%. For details on the tomography and data analysis in terms of structural and mechanical properties, see [28,29,44]. The model preparation requires only little pre-processing effort. Disconnected regions—made visible by gray color—bear no loads and are removed from the model. In order to detect these isolated regions, we make use of a seed-fill algorithm [47]. In a second step, we extracted a sub-sample from the full model that is small enough to be analyzed on a computer in a reasonable time and large enough to carry all characteristic information of the microstructure (see Figure 6b).

**Figure 7:**
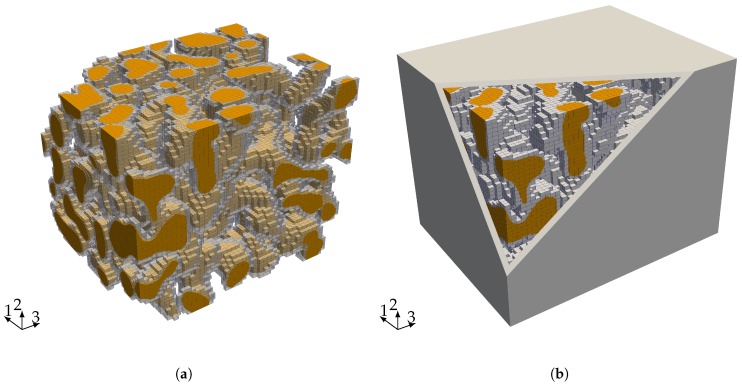
(**a**) shows the discretization (grey) with 72,465 finite cells and the microstructure (orange) of the sample and (**b**) the sample embedded in the window.

We discretize the sub-sample by finite cells where each cell contains four voxels per direction—see Figure 7a. The refinement of the spatial discretization is carried out by increasing the polynomial degree of the shape functions (*p-refinement*) and not by reducing the element size (*h-refinement*). The application of the window method requires to embed the sample of nanoporous gold into a so-called window as depicted in Figure 7b. Determining all elastic properties of the microstructure requires solving different load-cases. The window serves to apply the corresponding displacement boundary conditions not directly to the microstructure but to the window and by this lowers the influence of the boundary conditions. The window layer itself is composed of one layer of finite cells and is adjustable in thickness to model different types of boundary conditions. For a large window thickness, the boundary conditions resemble a Neumann boundary, while, for a small window thickness, they correspond to Dirichlet boundary conditions.

By changing the window thickness ($t_{w}\in \left\{0.357\;\mu m,0.714\;\mu m,1.072\;\mu m,1.423\;\mu m\right\}$) and its material properties, the surroundings of the sub-sample are modeled in a self consistent way and different results are obtained for different sizes of the window. The procedure regarding how to adjust the material properties is explained in more detail in Section 3.3. The material properties of the bulk gold material applied in the simulations chosen according to [15] are given in Table 1.

**Figure 8:**
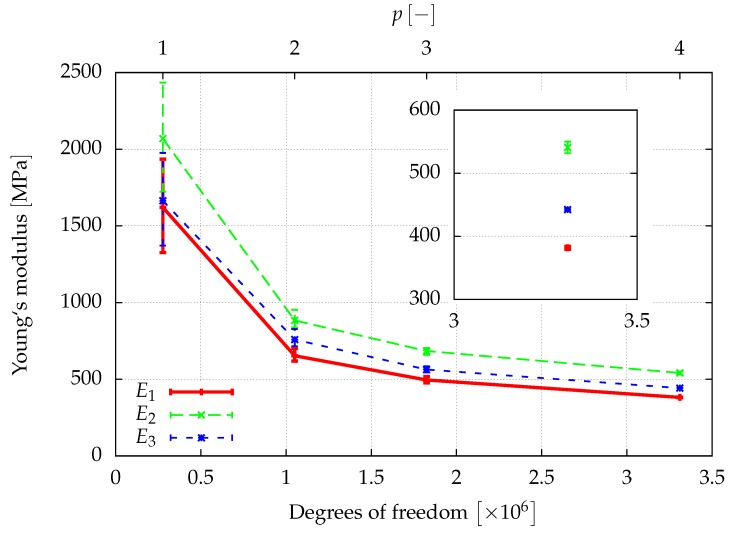
The spatial resolution of the discretization is improved by increasing the polynomial degree p=1,…,4 of the shape functions of the cells. The error bars show the influence of the window thickness.

**Figure 9:**
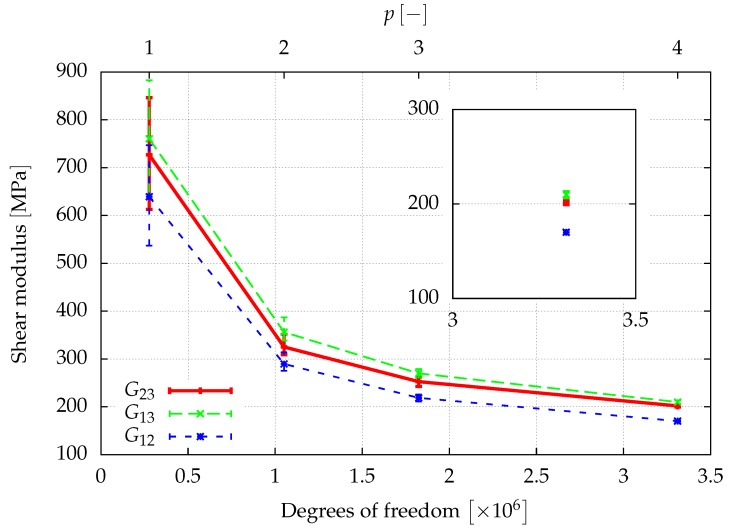
The spatial resolution of the discretization is improved by increasing the polynomial degree p=1,…,4 of the shape functions of the cells. The error bars show the influence of the window thickness.

**Figure 10:**
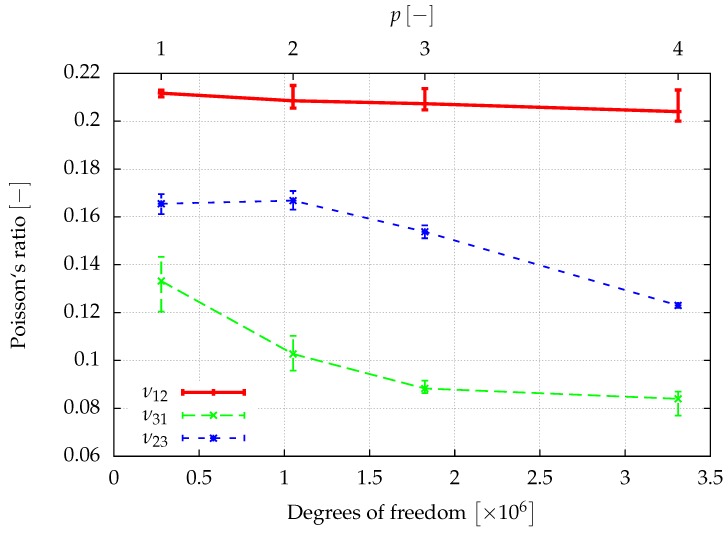
The spatial resolution of the discretization is improved by increasing the polynomial degree p=1,…,4 of the shape functions of the cells. The error bars show the influence of the window thickness.

The window method allows for finding the full anisotropic material properties of the sample as explained in Section 3.3. Assuming an orthotropic material behavior, the directional Young’s moduli, $E_{1},E_{2},E_{3}$, the shear moduli, $G_{12},G_{23},G_{13}$, and the Poisson ratios $\nu_{12},\nu_{23},\nu_{31}$, can be determined from a least squares fit of the obtained elasticity matrix. The results of a first investigation of the elastic properties are given in Figure 8, Figure 9 and Figure 10, where the convergence of the elastic parameters against the degrees of freedom for a uniform increase of the polynomial degree of the shape functions of all cells is plotted. The error-bars include the results obtained for different sizes of the window. With an increasing spatial resolution, the differences in the results obtained for different window sizes are more or less reduced and will be neglected in the further considerations. In summary, the obtained results are in the same range of the data already presented in [46] for a sample of the same size.

**Figure 11:**
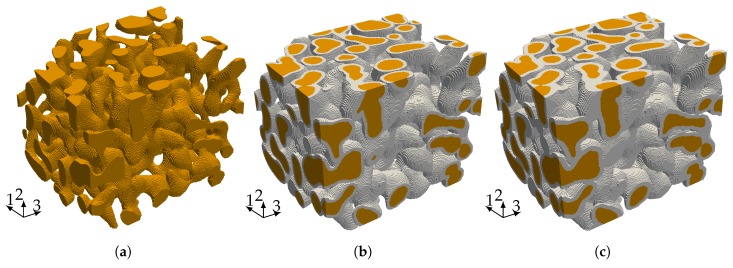
(**a**) sub-sample of a size of 3.57μm×3.57μm×4.6μm; (**b**,**c**) depict the sub-sample after applying a coating of $t_{c}=4\left[\mathrm{voxel}\right]$, resp. $t_{c}=6\left[\mathrm{voxel}\right]$ to the voxel model which corresponds due to the not equal voxel spacing to a coating thickness between $t_{c}=71.44\;nm-92\;nm$, resp. $t_{c}=107.16\;nm-138\;nm$.

For our second investigation, we apply a homogeneous polymer coating of $t_{c}=4\left[\mathrm{voxel}\right]$ (Figure 11b), resp. $t_{c}=6\left[\mathrm{voxel}\right]$ (Figure 11c) to the voxel model depicted in Figure 11a. The voxel spacing is not equal in each direction, so the coating thickness varies from $t_{c}=71.44\;nm$ to 92nm, resp. from $t_{c}=107.16\;nm$ to 138nm. A Cartesian mesh with 85,388, respectively 117,683 cells, is generated for the composites in the same fashion as for the nanoporous gold microstructure. The interface between the different materials is not resolved by cell interfaces, but considered during the integration of the stiffness matrix. To this end, we apply the adaptive Gaussian quadrature as explained in Section 3.1 to capture the material interface by means of an octree refinement.

For this study, we assume the properties of epoxy, which will also serve as starting values for the calibration of the polymer properties of PPy in Section 4.2. The material properties for the polymer used in the second investigation are taken from [46] and listed in Table 2.

**Figure 12:**
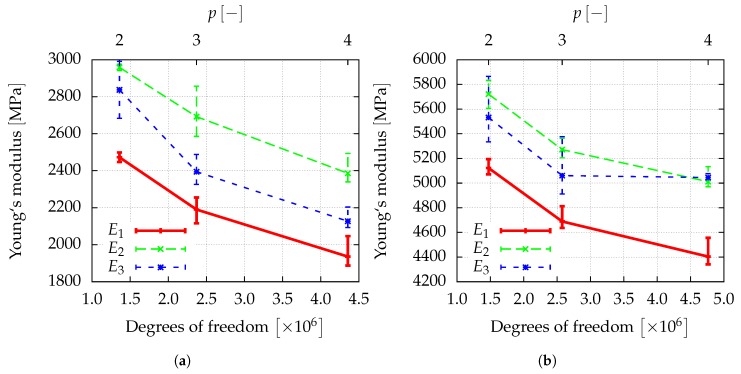
Different Young’s moduli of the gold–polymer composite with (**a**) $t_{c}=71.44\;nm-92\;nm$ and (**b**) $t_{c}=107.16\;nm-138\;nm$. The error-bars indicate the range of the results obtained for different sizes of the window.

Figure 12a,b show the convergence of the macroscopic elastic properties of the composite obtained from homogenization for a *p*-refinement, where p=2,3,4.

**Figure 13:**
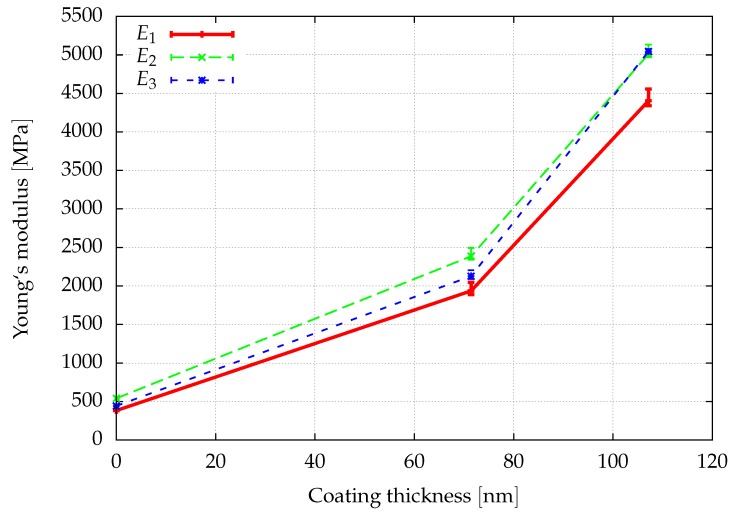
Comparison of the effect of the coating thickness on the Young’s moduli in the 1,2,3-direction.

By comparing the Young’s modulus for the finest discretization (p=4) in Figure 13, it is observed that the application of a polymer layer significantly increases the stiffness of the composite by up to a factor of 10, although the thickness of the polymer coating is below the ligament diameter and the Young’s modulus is only 6% of gold.

**Figure 14:**
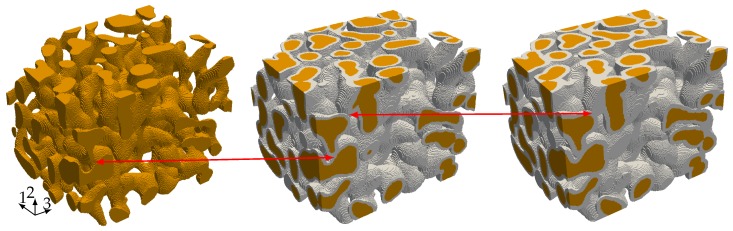
New connections formed by the coating layer.

If the coating was not a polymer but had the bulk properties of gold, one could simply estimate that, under bending dominated deformation of cylindrical ligaments [15], the geometrical increase in stiffness would scale with the area moment of inertia by a factor of $\frac{{\left(r+t_{c}\right)}^{4}}{r^{4}}\approx 6.71$. In tensile loading conditions, the increase in geometrical stiffness would scale by the area of the ligament $\frac{{\left(r+t_{c}\right)}^{2}}{r^{2}}\approx 2.59$. When considering that the real coating is a polymer which is more compliant than gold, the real stiffening effect would be even less than given by these two estimates. However, both scaling relations still underestimate the real increase in stiffness. This indicates that the dominant stiffening effect of the additional coating has other origins among which the forming of new connections between neighboring ligaments is one mechanism—see Figure 14.

### 4.2. Investigation of Elastoplastic Properties

**Figure 15:**
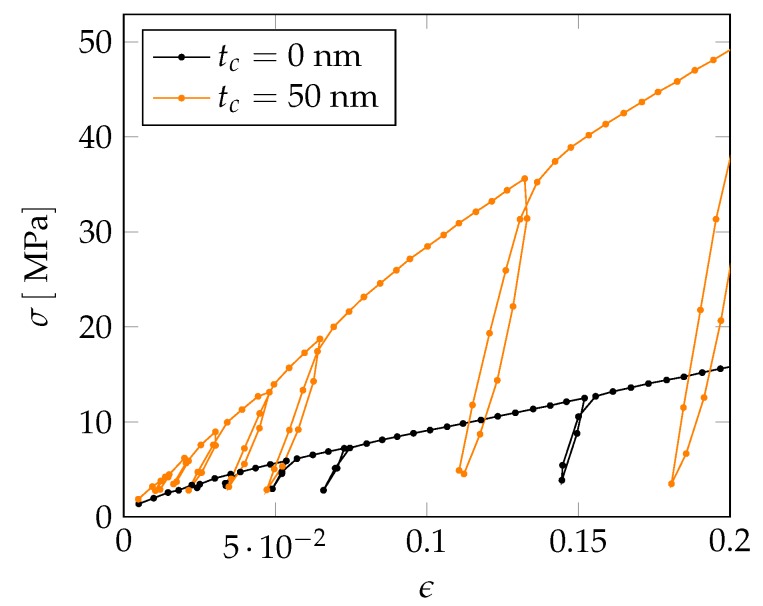
Experimentally obtained true stress–strain curves of non-coated nanoporous gold $t_{c}=0\;nm$ and NPG-PPy nanocomposite (PPy coating thickness of $t_{c}=50\;nm$). The ligament diameter for both samples is $d_{lig}=220\;nm$.

In a next step, we extend our investigations to the nonlinear regime by also considering the plastic behavior of the NPG-PPy nanocomposite. In order to relate the computations to experiments, we consider experimentally obtained true stress–strain curves on nanoporous gold samples with a ligament diameter of $d_{lig}=220\;nm$ and NPG samples coated by a PPy layer with a thickness of $t_{c}=50\;nm$—see Figure 15. An important assumption that allows us to apply the tomography data of a NPG sample with $d_{lig}=421\;nm$ to a smaller ligament diameter is the self-similarity of the NPG which holds for structures with a ligament diameter $d_{lig}\geq 100\;nm$. The self-similarity of NPG has been intensively investigated for a large range of ligament sizes in [28,44].

The deformation behavior of the non-coated NPG under compression is comparable to previous studies of NPG specimens with similar ligament size [8,9,33]. Here, we also find an absence of the initial elastic regime and an early yielding along with weak work hardening at higher strains. In contrast, an NPG-PPy nanocomposite exhibits the enhanced flow stress and work hardening, although the elastic-plastic transition is still ill defined. The effective elastic moduli as inferred from the first unloading cycles for both samples differ by less than 100MPa—see Table 3. The presence of the $t_{c}=50\;nm$ thick PPy layer marginally stiffens NPG, by about 20%. The influence of the polymer coating on the plastic flow, however, is more significant. For instance, at 0.15 strain, there is a nearly 4-fold increase in the flow stress of the nanocomposite.

In order to fit the numerical stress–strain curve to the one of the NPG-composite, we follow a two-step approach. In a first step, we adjust the properties of the nanoporous gold such that we meet the experimental curve of nanoporous gold. In a second step, we take the fitted properties of the nanoporous gold and apply bulk polymer material properties of epoxy as starting values and adjust the polymer parameters until we meet the experimental NPG-composite stress–strain curve.

The values for the macroscopic yield stress as well as the macroscopic elastic modulus determined from the first unloading are given in Table 3. To have a unique definition for the yield stress, we took the offset yield point where 0.2% plastic deformation occurs.

In order to simulate the coating, we added a homogeneous layer of polymer with $t_{c}=107.16\;nm-138\;nm$—see Figure 11c. These values correspond to the same ratio $t_{c}$ vs. $d_{lig}$ as for the experimentally tested samples. As a starting point, we take bulk values for the material properties from [46] which are listed in Table 4 and apply them to the sample of nanoporous gold that is shown in Figure 11a.

In order to analyze the anisotropic behavior of the nanoporous gold, we apply three different compressive loadings in 1-, 2- and 3-direction by clamping the lower side of the sample and prescribing a displacement on the upper side. The discretization consists of $n_{c}=$ 130,114 cells with a size of 71.44nm×71.44nm×92nm.

**Figure 16:**
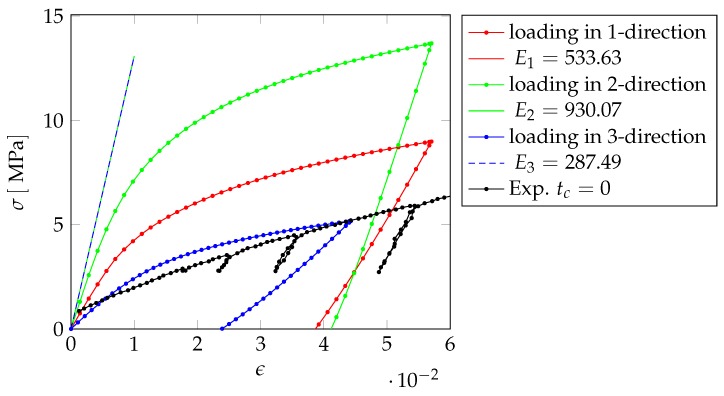
Comparison of the numerically obtained stress–strain curve with experiments using bulk material parameters.

Figure 16 shows the comparison of the numerical results of this first investigation to the experimental results.

Since the slopes of the elastic unloading fit best for the 1-direction and also fit to the results for the homogenization, we consider this loading direction for our further investigations.

**Figure 17:**
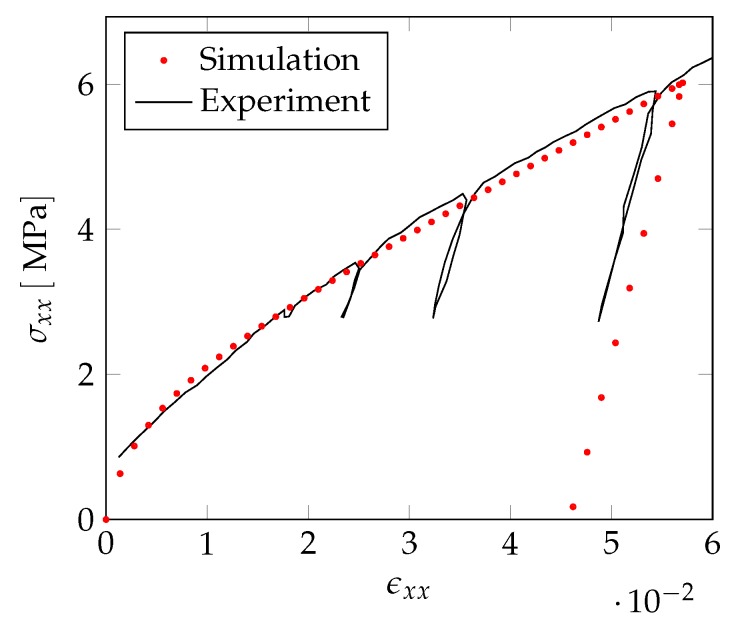
Comparison of experimental and numerical stress–strain curve after fitting the material parameters.

In a next step, we change the initial yield stress $\sigma_{0}$ and the hardening modulus of the gold, such that the numerical stress–strain curve fits the experimental one. Figure 17 shows the stress–strain curve after fitting the material parameters of the gold. The Young’s modulus of the sample is E=451.56MPa and the initial yield stress $\sigma_{0}=1.48\;M\mathrm{Pa}$, yielding a sufficiently close fit to the experimental values listed in Table 3.

The fitted material parameters for gold are given in Table 5. The initial yield stress compares very well with the values determined by [10] for ligaments of 200nm which ranged from 60MPa to 100MPa, but the hardening modulus is much higher by about an order of magnitude compared to the values found by [15,28] who used a work hardening rate of 1000MPa.

In a next step, we analyze a coated sample using the material parameters of the nanoporous gold obtained from the fit together with material parameters for the polymer for which we use properties of epoxy as starting values—see Table 6.

**Figure 18:**
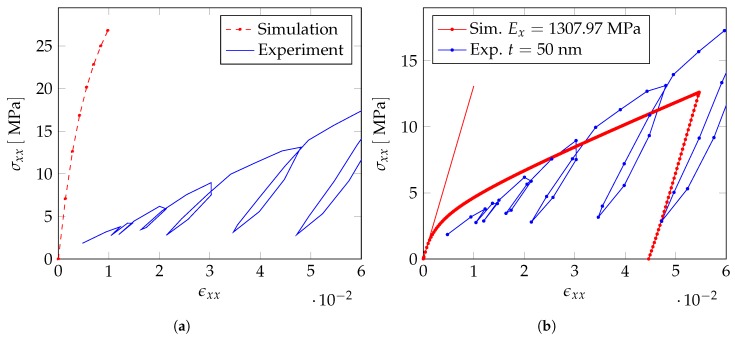
(**a**) Comparison of the experimentally obtained stress–strain curve with the numerical stress strain curve using the adjusted material parameter of gold and epoxy material properties for the polymer coating as starting values for fitting procedure and (**b**) numerical and experimental stress–strain curve after adjusting the material parameters of the polymer.

Applying these material parameters, the polymer has a much more pronounced stiffening effect on the stress–strain curve than determined from the experiments—see Figure 18a.

Therefore, in a next step, we also adjust the material parameters of the polymer in our numerical computations, such that the stress–strain curve fits to the experiments—see Figure 18b. For the fitted material parameters, which are given in Table 7, the computed Young’s modulus of the macroscopic sample is E=1307.71MPa and the initial yield stress is $R_{p0,2}=3.06\;M\mathrm{Pa}$. Both are still slightly higher than the values measured from the experimental stress–strain curve. In order to fully align the numerical computations with the experiments, a further reduction of the Young’s modulus and the initial yield stress of the polymer coating is required. However, the further reduction of these material parameters yields to a very poor conditioning of the resulting stiffness matrix which causes convergence problems of the Newton–Raphson method.

Our numerical investigations of nanoporous gold samples with a ligament diameter of $d_{lig}=421\;nm$ and a polymer coating of $t_{c}=107.16\;nm-138\;nm$ indicate an enormous increase of the macroscopic stiffness by a factor of ten if a polymer coating such as epoxy resin with bulk material values could be applied—see Figure 13. In line with our expectations from the experimental results, the increase in stiffness entailed by coating the gold ligaments with the softer polymer such as PPy is ten times smaller. Our numerical computations capture the stress–strain behavior of the NPG-PPy nanocomposite and predict the mechanical properties of PPy. The Young’s modulus and yield stress of PPy which need to be applied in the numerical computations in order to match the stress–strain curve of the experiments have been found to be 200MPa and 2MPa, respectively. The values match well to the experimental data available in the literature, where the elastic moduli have been reported in the range of 100–4300 MPa [48,49,50,51,52,53,54] and the yield strength between 2.4–5.3 MPa [49]. Significant variations in the mechanical parameters of the bulk PPy in the literature have been attributed to the sensitivity of the PPy morphology and structure to synthesis conditions [55]. Among other factors, a type of the dopant counterions used for electropolymerization can strongly affect PPy mechanical characteristics. For the perchlorate anion employed as a dopant during synthesis of PPy in our work, the reported values of elastic modulus are in the range 100–300 MPa [48], and agree well with the predictions from our simulations. The good agreement underlines the high sensitivity of the approach for calibration of the material parameters for PPy using the numerical model. Starting the calibration with much larger values taken from bulk epoxy, the solution rapidly converged to the much lower and much more realistic values of PPy within a few iterations. In this way, there is now a set of material parameters for PPy available that includes the yield stress and hardening rate for the given synthesis conditions on NPG.

## 5. Conclusions

In this paper, we investigated the potential stiffening effect of an infiltrated epoxy resin and an electrolytic applied polypyrrole polymer on the elastic and elastoplastic properties of NPG-composites. For the infiltrated epoxy resin, we could determine the anisotropic elasticity matrix by employing the window method and computing a full set of transversal isotropic material properties by a least-square fit. By this procedure, we could show that the Young’s moduli increase significantly due to new connections formed between the ligaments when a coating is applied. The obtained results are in good agreement with already existing data. In a second investigation, we analyzed the influence of an electrolytic applied polypyrrole coating by direct comparisons with experiments on NPG with a ligament size of $d_{lig}=200\;nm$. Due to the self similarity of these structures, we could reuse our geometric model with a ligament diameter of $d_{lig}=421\;nm$. After fitting the material properties of a pure NPG sample to meet the experimentally obtained stress–strain curve, we created a numerical NPG-composite by manipulating the voxel model. After applying a coating with the same $t_{c}$ vs. $d_{lig}$ ratio as was used for the experiments, we could analyze the properties of an electrolytic applied PPy coating. Due to the sensitivity of PPy with respect to its synthesis condition, the focus was on inverse computations that served to determine the elastoplastic properties of PPy. The final numerically determined parameters for PPy are in good agreement with already existing data of PPy obtained under similar synthesis conditions. With this, it is demonstrated that the Finite Cell Method is an efficient alternative to common Finite Element approaches to predict the elastic-plastic deformation behavior of NPG and polymer coated NPG. This approach has the big advantage that the model can be set-up in a straightforward way based on tomography data without the usual challenge to discretize the complex structure of NPG by a surface conforming mesh. 

## Figures and Tables

**Table 1:** Material properties of gold applied for the homogenization.

	Parameter	Value	Unit
Young’s modulus	*E*	81	GPa
Poisson’s ratio:	ν	0.42	

**Table 2:** Elastic material properties of polymer coating applied for the homogenization.

	Parameter	Value	Unit
Young’s modulus	*E*	4.7	GPa
Poisson’s ratio	ν	0.3	

**Table 3:** Parameter of the macroscopic stress–strain curve for experiments on pure and coated nanoporous gold showing a stiffening effect of the polymer by a factor of 1.19.

	$R_{p0,2}$	*E*
Nanoporous gold (NPG)	1.51MPa	395.95MPa
Composite	2.66MPa	473.12MPa

**Table 4:** Bulk material properties of gold.

	Parameter	Bulk Value	Unit
**Gold**			
Young’s modulus	*E*	81	GPa
Poisson’s ratio	ν	0.42	
Initial yield stress	$\sigma_{0}$	700	MPa
Hardening modulus	*h*	1000	MPa

**Table 5:** Fitted material properties of gold.

	Parameter	Fitted Value	Bulk Value
**Gold**			
Young’s modulus	*E*	81GPa	81GPa
Poisson’s ratio	ν	0.42GPa	0.42GPa
Initial yield stress	$\sigma_{0}$	90MPa	700MPa
Hardening modulus	*h*	11,000MPa	1000MPa

**Table 6:** Bulk material properties of the polymer coating.

	Parameter	Bulk Value
**Polymer**		
Young’s modulus	*E*	4.7GPa
Poisson’s ratio	ν	0.3GPa
Initial yield stress	$\sigma_{0}$	80MPa
Hardening modulus	*h*	10MPa

**Table 7:** Fitted material properties of gold and polymer coating and their starting values.

	Parameter	Starting Value	Fitted Value
**Gold**			
Young’s modulus	*E*	81GPa	81GPa
Poisson’s ratio	ν	0.42GPa	0.42GPa
Initial yield stress	$\sigma_{0}$	700MPa	90MPa
Hardening modulus	*h*	1000MPa	11,000MPa
**Polymer**			
Young’s modulus	*E*	4.7GPa	200MPa
Poisson’s ratio:	ν	0.3GPa	0.3GPa
Initial yield stress	$\sigma_{0}$	80MPa	2MPa
Hardening modulus	*h*	10MPa	10MPa

## Abbreviations

The following abbreviations are used in this manuscript:

FCM	Finite cell method
NPG	Nanoporous gold
RVE	Representative volume element
TVE	Testing volume element
PPy	Polypyrrole
